# Feasibility, Acceptability, and Preliminary Efficacy of a Remotely-Delivered Health Coaching Intervention for Young and Middle-Aged Cancer Survivors: A Phase II Pilot Study

**DOI:** 10.1007/s13187-025-02770-4

**Published:** 2025-11-06

**Authors:** Lauren Tetmeyer, Nicholas Farkas, Garrett M Steinbrink, Katherine Mellen, Erin Litton, Jessica Gorzelitz, Mary C. Schroeder, Chooza Moon, Lucas J. Carr

**Affiliations:** 1 Department of Health, Sport, and Human Physiology, University of Iowa, Iowa City, USA; 2 Department of Pharmacy Practice and Science, University of Iowa, Iowa City, USA; 3 College of Nursing, University of Iowa, Iowa City, USA; 4 Holden Comprehensive Cancer Center, University of Iowa, Iowa City, IA USA

**Keywords:** Lifestyle, Survivorship, Remote, Health coaching, Feasibility

## Abstract

Cancer survivors living in rural areas have poorer health outcomes due in part to poorer access to survivorship programs. This study evaluated the feasibility, acceptability, and preliminary efficacy of a scalable, remotely-delivered health coaching lifestyle intervention for cancer survivors. In this phase II pilot study, we recruited young and middle-aged adult cancer survivors (18–64 years) to participate in a remotely-delivered eight-week lifestyle behavior change intervention that included remote health coaching sessions (administered by trained student health coaches), remote health education videos, and a wearable activity/sleep monitor. Feasibility was measured by enrollment, retention, and attrition rates, while acceptability was assessed via a post-intervention survey. Preliminary efficacy was evaluated by changes in psychosocial mechanisms, health behaviors, and cancer-related health outcomes. Our enrollment rate (49%) fell just short of our target (50%). Our retention rate (83%) exceeded our target rate of 75%. Adherence rates also exceeded targets (75%) with 99% of remote health coaching sessions attended, 84% of educational videos viewed, and activity monitors worn on 87% of all intervention days. Acceptability rates were also high with most participants reporting the program was beneficial (90%) and that they would recommend it to others (87%). We observed medium-large effects for several secondary/exploratory outcomes including behavioral regulation skills (Cohen’s d = 2.04), fatigue (d = 0.86), and global health scores (d = 0.99). No pre-post changes were observed for physical activity, diet, or sleep behaviors. Findings suggest it is feasible to deliver an acceptable remote lifestyle intervention to young and middle-aged cancer survivors. Improvements in targeted psychosocial and cancer-related health markers are promising and support the need for a larger, controlled follow-up study. These findings support the feasibility of delivering a remote, lifestyle behavior change program to young and middle-aged cancer survivors.

## Key Messages regarding feasibility

• Prior to this study, uncertainties existed regarding the feasibility of delivering a completely remote health coaching intervention to cancer survivors.

• We found that our team was able to deliver the intervention as intended to cancer survivors from seven different states. We observed a low attrition rate and high retention and adherence rates.

• These findings suggest the intervention was designed effectively to reach the target population.

## Introduction

Over 18 million Americans today live with cancer [1, 2]. With advances in cancer treatment and a growing aging population, this number is projected to rise. Adults living with cancer often experience long-term side effects of both the cancer and its treatment, including weight gain, physical impairments, fatigue, sleep difficulties, and reduced quality of life [3–5]. Improving the quality of survivorship has been identified as an important but understudied area of research [6, 7]. The American Cancer Society and the National Comprehensive Cancer Network recommend all cancer survivors maintain a healthy lifestyle that includes being physically active, avoiding sedentary behavior, getting adequate sleep, following a healthy eating pattern, and maintaining a healthy weight [8, 9]. Strong evidence suggests that engaging in these lifestyle behaviors can improve cancer-related health outcomes, such as fatigue, quality of life, and recurrence risk [10, 11].

Cancer survivor’s adherence to these recommended lifestyle behaviors remain low [12]. Specifically, up to 70% of survivors fail to meet physical activity guidelines [13], and approximately 85% consume fewer than five servings of fruits and vegetables per day [12]. Sleep disturbances are also common, with up to 40% of survivors experiencing poor sleep quality five years post-diagnosis [14]. National organizations have called for comprehensive survivorship programs that include support for adopting healthy behaviors post-treatment [15, 16]. However, the implementation of such programs is hindered by workforce limitations, limited reimbursement structures, and fragmented systems of care [17–20]. Additionally, survivors report individual level barriers that limit healthy lifestyles including cancer-related fatigue, stress, time constraints, limited social support, and lack of knowledge about effective behavior change strategies [21].

Survivors of cancer living in rural areas experience higher mortality rates than survivors in urban areas [22]. Poorer outcomes among rural survivors are thought to be associated with poorer adherence to recommended lifestyle behaviors. Rural cancer patients often face unique challenges in receiving care due to limited availability of cancer support providers, transportation barriers, financial issues, and limited access to clinical trials [23]. There is a need for effective interventions that can be delivered at scale to meet the needs of survivors in both urban and rural areas.

Theory-guided, personalized interventions are effective at improving lifestyle behaviors [24] but are often limited by their scalability. Health coaching is a personalized intervention approach that is goal-oriented, client-centered and health-focused [25]. Prior studies among generally healthy and chronic disease populations suggest health coaching is effective for improving physical activity, diet, and sleep behaviors [26, 27]. While preliminary findings suggest health coaching may also benefit adults living with cancer [28, 29], few studies have explored the efficacy of health coaching among survivors of cancer. Further, few health coaching interventions have been delivered completely remotely to cancer survivors.

This study (known as Vitalis Cancer) aimed to evaluate the feasibility, acceptability, and pre-post changes of an 8-week, remotely delivered health coaching intervention designed to improve: (1) targeted psychosocial mechanisms of action (behavioral regulation skills, affective attitudes, health habits, and identity); (2) health behaviors (physical activity, diet, sleep); and (3) cancer-related health outcomes (physical function, anxiety, depression, fatigue, and pain) among young and middle-aged adult cancer survivors.

## Methods

### Study Design and Overview

The Vitalis Cancer study was a single-arm, remotely delivered pilot trial to assess the feasibility, acceptability, and pre-post changes of a lifestyle behavior change intervention among cancer survivors. The study was conducted in Iowa City, Iowa, between January and September 2023. The intervention was designed using the Obesity-Related Behavioral Intervention Trials (ORBIT) model [30] and informed by the Multi-Process Action Control (M-PAC) Theory [31]. Additionally, this study used a remote delivery model to increase accessibility. To address the scalability limitations that often plague individual level interventions, health coaching was delivered by trained students using a service-learning model. The intervention targeted multiple health behaviors—physical activity, diet, and sleep—simultaneously, reflecting their interrelated nature and their combined influence on quality of life and long-term health outcomes. The research team included professionals with expertise in behavioral science, lifestyle behavior change, cancer survivorship, and health coaching. Their expertise guided the tailoring of the intervention to address the barriers reported by cancer survivors, such as lack of time, support, and health behavior knowledge. This study was retrospectively registered at ClinicalTrials.gov on 5/30/2025 (NCT07026214).

## Participants

Eligible participants were young and middle-aged adult cancer survivors aged 18–64 years, 3–60 months post-treatment, and not meeting recommendations for physical activity, diet, or sleep. Participants had to express interest in improving at least one health behavior. Exclusion criteria included current cancer treatment, metastatic disease, pregnancy or plans for pregnancy, and no access to Zoom or a smartphone. These exclusions were applied to ensure intervention appropriateness, safety, and comparability of outcomes.

## Intervention and Theoretical Framework

Intervention design was guided by the Multi-Process Action Control (M-PAC) Theory, which is a meta theory that suggests behavior change occurs through intention formation, adoption, and maintenance processes of motivation [31]. This theory is especially well-suited to lifestyle interventions, as it targets modifiable constructs such as self-regulation of behaviors. The M-PAC theory also supports behavior change promotion through the application of demonstrated behavior change techniques that align well with health coaching principles including goal setting, self-monitoring, problem-solving, and action planning. By targeting these psychosocial mechanisms, M-PAC aligns with the goals of survivorship care and supports sustained behavior change in diverse populations. In addition to health coaching, the intervention included health education, and access to a wearable activity/sleep monitor.

### Health Coaching

The health coaching intervention was delivered by students through a service-learning model, an educational practice that trains students to deliver services to community members in need [32]. The service-learning model was selected due to its potential to scale individualized interventions to larger population and reduce health disparities. The students were all enrolled in a health coaching training program that has been approved by the National Board for Health and Wellness Coaching (NBHWC). The health coaching training program is led by two full-time faculty members who are also board-certified health coaches through the NBHWC. As part of the training program, and prior to delivering the health coaching sessions to participants in the study, all health coaching interns completed four semester-long undergraduate courses on physical activity, nutrition, lifestyle medicine, and health coaching. Student’s health coaching proficiency was assessed by faculty instructors at four timepoints: lifestyle medicine class (30-minute session), health coaching class (30-minute session), in a practical skills assessment (30-minute session), and in the health coaching internship (two 30-minute sessions). Course directors assess the quality of health coaching sessions using an NBHWC approved rubric. The grading rubric includes 18 observable skills including mindful presence, empathy, active listening, use of open-ended questions, leveraging change talk, goal setting, and time management. To pass the practical skills exam and enroll in the health coaching internship, where student health coaches deliver this intervention, students must score an 80%. All student health coaches in this study passed this practical skills exam.

As part of the intervention, each participant received five coaching sessions led by a student health coach. All coaching sessions were delivered remotely using a HIPAA-compliant telehealth health coaching platform, Healthie (Healthie Inc., New York, NY, USA). The first session was 90 min long, followed by four 15–30-minute follow-up sessions. Each coaching session followed a standardized script, developed by the health coaching program directors, and included several evidence-based behavior change techniques including prompting goal-setting, barrier identification, problem-solving, action planning, and relapse prevention [33].

### Health Education

Participants received access to three, 8–10-minute health education videos focused on physical activity, diet, and sleep. The videos were developed by content experts on the team (LC, KM, CM) and were designed to provide participants with introductory information on the associations between lifestyle behaviors and cancer-related health outcomes, lifestyle behavior recommendations for cancer survivors (e.g., 150 min of moderate PA per week, ≥ 5 servings of fruits/vegetables per day, ≥ 7 h of sleep/night), and tips for achieving the recommendations. Health education videos were made available to participants remotely through the Healthie telehealth platform and participants were encouraged to watch the videos on their own time.

### Self-Monitoring 

Participants were mailed a wrist-worn Fitbit Inspire 2 activity/sleep monitor and were encouraged to wear the monitor day and night to self-monitor their physical activity and sleep behaviors. A valid wear day required ≥ 100 steps, consistent with a strategy our team used in a previous study [34]. While this approach provided accurate behavioral data, participant burden was addressed via detailed informed consent and secure data handling. Privacy concerns related to Fitbit login sharing were mitigated with temporary credentials and explained during onboarding.

## Outcomes and Measures

Feasibility metrics included enrollment rate (% eligible participants allocated to the intervention), retention (% allocated to intervention who completed study), and intervention adherence rates (% participants who completed health coaching, health education, self-monitoring intervention functions) with thresholds based on similar pilot studies led by members of our team (≥ 50% enrollment, ≥ 80% retention, ≥ 70% adherence) [35]. Acceptability was assessed post-intervention via Likert-scale surveys with adequate acceptability defined as ≥ 75% of respondents reporting satisfaction or agreement. These thresholds were drawn from prior digital health intervention literature and a priori study goals [36].

### Demographics and Clinical Outcomes

 All assessments were collected remotely. At baseline, participants completed an 11-item online survey (Qualtrics, Provo, UT) collecting age, weight, height, education, employment status, sex, ethnicity, race, household income, marital status, and self-reported cancer type. Height and weight were self-reported and used to calculate body mass index (BMI, kg/m²). We used the Rural Health Information Hub “Am I Rural?” Tool to determine whether participants were living areas considered to be rural and/or in medically underserved areas [37]. We used Google Maps to calculate the distance from the university hospital where the intervention was delivered to participants’ location of residence.

### Psychosocial Mechanisms

 At baseline and post-intervention, participants completed a 24-item online survey adapted from the Multi-Process Action Control (M-PAC) Questionnaire [38]. Measured constructs included affective and instrumental attitudes, perceived capability and opportunity, behavioral regulation skills, health habits, and health identity. Responses were collected on a 5-point Likert scale (1 = strongly disagree, 5 = strongly agree). While higher scores generally reflect greater alignment with health-supportive constructs (e.g., stronger identity or skills), each domain measures a distinct theoretical construct and should be interpreted independently.

### Physical Activity Behavior

 Physical activity was measured using a thigh-worn activPAL monitor (Pal Technologies, Glasgow, UK) which was mailed to participants with written instructions.[39] Participants wore the monitor 24 h/day for seven days at baseline and post-intervention using waterproof dressings and recorded removal times in a mailed log. Valid days were defined by ≥ 20 h of wear [40]. Outcomes included daily steps, cadence, stepping duration, and sedentary behavior. Data were included for participants with at least four valid days.

### Dietary Behavior

 Diet was assessed using the National Cancer Institute’s (NCI) Automated Self-Administered 24-Hour Dietary Assessment Tool (ASA24) [41–43]. Participants completed one 24-hour dietary recall online. Nutrient and food group data were processed using NCI’s open-source code to calculate total daily kilocalories and the Healthy Eating Index (HEI), a measure of diet quality [44].

### Sleep Behavior

Sleep was measured using an ActTrust2 actigraph (Condor Instruments, São Paulo, Brazil), worn on the non-dominant wrist for seven consecutive days. Participants pressed a button on the monitor to log sleep/wake times and tracked non-wear time with a daily log. Valid data required ≥ 10 h of daily wear. Outcomes included total sleep time, sleep efficiency, and wake after sleep onset.

### Cancer-Related Health Outcomes 

Participants completed the 29-item PROMIS Global Health Scale remotely (Qualtrics) at baseline and post-intervention, measuring domains such as fatigue, physical function, sleep disturbance, and social participation. Each domain consisted of four items rated on a five-point Likert scale. Scores were averaged per domain for analysis. These assessments allowed for comprehensive evaluation of well-being in cancer survivorship [45].

#### Statistical Analysis

Feasibility and acceptability were the primary outcomes. Feasibility was defined a priori using benchmarks common in pilot trials (≥ 50% enrollment rate, ≥ 80% retention rate, ≥ 70% intervention adherence rate). Acceptability was defined as > 75% agreement on satisfaction surveys. Feasibility and acceptability outcomes were analyzed using descriptive analyses. As secondary/exploratory outcomes, preliminary efficacy was assessed using paired t-tests in targeted psychosocial (attitudes, perceived capability, perceived opportunity, behavioral regulation skills, habit, identity), behavioral (physical activity, diet, sleep), and cancer-related health outcomes (physical function, anxiety, depression, fatigue, sleep disturbance, social roles, pain interference, pain intensity, global health). Cohen’s *d*, or the standardized mean difference, was calculated to estimate the effect size of the observed change in secondary outcomes of interest, with effect sizes < 0.2, 0.2–0.49, 0.5–0.79, and ≥ 0.8 considered trivial, small, medium, and large, respectively [46]. All analyses were conducted using Stata/MP v15 (StataCorp, College Station, TX).

## Results

Study completers (*n* = 30) had an average BMI of 31.3 ± 5.8 kg/m², were predominantly female (80%), White (93%), and non-Hispanic (96%), and most had a college degree or higher (76%). Breast and hematological cancers were the most common diagnoses (60%). Participants were recruited from seven different states, mostly in the Midwest region. More than a third of participants resided in rural areas and participants lived an average of 134 miles from the university where the intervention was delivered (Table 1).

**Table 1:** Participant demographic characteristics (*n* = 30)

Demographic Variable ​​	Mean (SD)​​ or %
Age (years)​​	42.0 (10.8)​​
Weight (lbs)​​	193.4 (36.6)​​
BMI​​ (kg/m^2^)	31.3 (5.8)​​
Educational Attainment​​ (% college graduate or higher)	76%
Employed (%)	60%
Sex ​​	
Female	24
Male	6
Ethnicity ​​(% Not Hispanic or Latino)	96%
Race​​ (% White)	93%
Household Income​​ (% >$75,000/year)	66%
Distance from Residence to Hospital (miles)	134.9 (268.8)
Residence in Rural Area? (% Yes)	36.7%
Residence in Medically Underserved Area? (% Yes)	13.3%
Cancer Type	
Breast​	36.7%
Hematological Oncology​	23.3%
Gastrointestinal	13.3%
Head and Neck​	13.3%
Bone​	10%
Gynecological​	3.3%​

### Feasibility

**Fig. 1:**
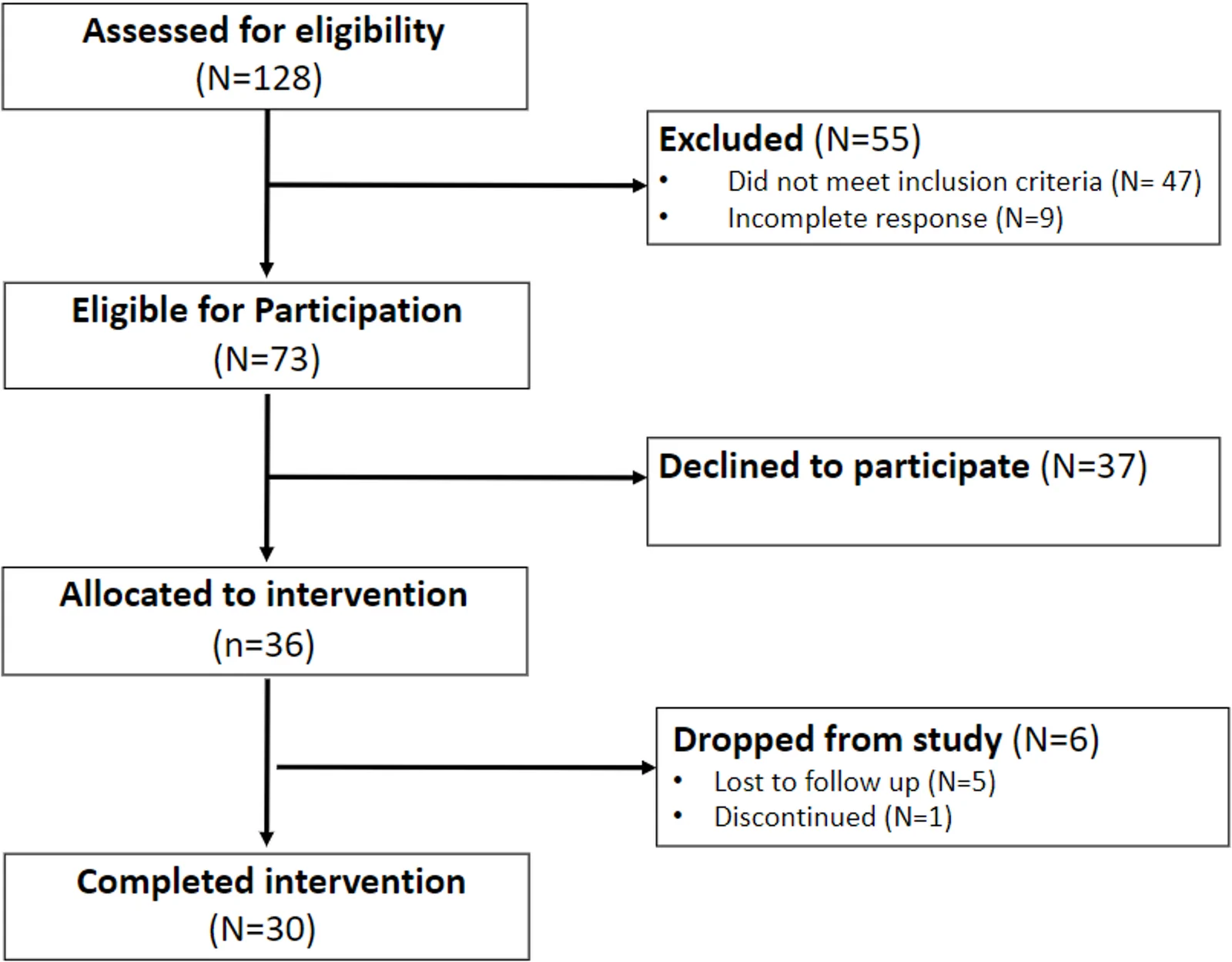
CONSORT diagram

Recruitment, retention, and completion details are available in the CONSORT diagram (Fig. 1). A total of 128 individuals completed an eligibility survey, of which 55 individuals (43%) were excluded for not meeting inclusion criteria (*n* = 47) or for other reasons (*n* = 9). A total of 73 individuals were found eligible to participate. A total of 37 of the 73 eligible participants (51%) declined to participate resulting in a 49% enrollment rate. Six of the 36 (16.7%) participants who were enrolled in the study and allocated to intervention did not complete the study resulting in an 83.3% retention rate. Participants completed 99% of scheduled health coaching sessions (148/150), viewed 84% of health education videos (76/90), and wore their activity/sleep monitor on 87% of days (1,255/1,442 days).

#### Acceptability 

Acceptability ratings were favorable: 87% of participants agreed or strongly agreed the program was enjoyable, 83% felt it was designed for people like them, 90% found it beneficial for their health, and 87% would recommend it to others. Satisfaction with specific features was > 75% for instructions (97%), health coaching (83%), Fitbit use (83%), program length (93%), and remote delivery (97%). Lower satisfaction was reported for the mobile app (Healthie) and educational videos (see Table 2).

**Table 2:** Minimum, maximum, and mean responses to participant satisfaction surveys (*n* = 30)

	Min	Max	Mean	SD
Please answer the following questions related to your level of satisfaction with this program (1 = Strongly disagree, 2 = Disagree, 3 = Neutral, 4 = Agree, 5 = Strongly agree)				
I enjoyed participating in this program.	3.0	5.0	4.40	0.71
I found this program to be helpful for improving my health.	2.0	5.0	4.40	0.76
I would recommend this program to others.	2.0	5.0	4.37	0.8
This program was designed for people like me.	2.0	5.0	4.23	0.88
Please rate how satisfied you were with each of the following program features (1 = Very dissatisfied; 2 = Dissatisfied; 3 = Neutral; 4 = Satisfied; 5 = Very Satisfied)				
Remote/Virtual Delivery of Program	3.0	5.0	4.63	0.66
Health Coaching	2.0	5.0	4.47	0.92
Length of the program (8 weeks)	2.0	5.0	4.37	0.76
Fitbit Activity/Sleep Monitor	3.0	5.0	4.23	0.78
Physical activity video(s)	3.0	5.0	3.87	0.76
Healthy Eating Video(s)	3.0	5.0	3.83	1.02
Sleep Video(s)	3.0	5.0	3.77	0.71
Healthie Coaching Mobile App	2.0	5.0	3.60	0.55

##### Secondary/Exploratory Efficacy Outcomes 

When evaluating pre-post changes in measured psychosocial mechanisms of action, we observed a large effect for behavioral regulation skills (d = 2.04), medium-sized effects for health habits (d = 0.52) and health identity (d = 0.71), and small effects for perceived capability (d = 0.41), behavioral intentions (d = 0.31), and affective attitudes (d = 0.23). All effects were in the expected direction. Trivial effects were observed for perceived opportunity (d = 0.01) and instrumental attitudes (d = −0.12) (see Table 3).

**Table 3:** Psychosocial constructs at baseline and post-intervention (*N* = 30)

Psychosocial Construct	Baseline Mean (SD)​	Post-Intervention Mean (SD)	Cohen’s d
Affective Attitudes (out of 15) ​	12.8 (1.3)​	13.1 (1.3)	0.23
Instrumental Attitudes (out of 15)​	14.5 (0.8)​	14.4 (0.8)	−0.12
Perceived Capability (out of 15)​	12.1 (1.4)​	12.7 (1.5)	0.41
Perceived Opportunity (out of 15)​	13.2 (1.5)​	13.2 (1.7)	0.01
Intentions (out of 15)​	12.5 (1.5)​	13.0 (1.7)	0.31
Behavioral Skills (out of 27)​	15.9 (4.7)​	23.8 (2.8)	2.04
Habits (out of 15)​	8.4 (2.1)​	9.5 (2.1)	0.52
Identity (out of 15)​	9.3 (1.8)​	10.6 (1.8)	0.72

High score indicates superior status for psychosocial constructs

When evaluating health behaviors, trivial effects were observed for targeted physical activity behaviors (see Table 4). A small effect in the expected direction was observed for daily kilocalories (d = −0.20). A small effect in the unexpected direction was observed for total HEI scores (d = −0.23). Trivial effects were observed for most targeted sleep behavior outcomes including total sleep time and sleep efficiency. A small and medium effect were observed for number of nightly awakenings (d = 0.27) and sleep onset latency (d = 0.72), respectively.

**Table 4:** Physical activity, diet, and sleep behaviors at baseline and post-intervention (*n* = 30)

	Baseline Mean (SD)	Post-Intervention Mean (SD)	Cohen’s d
Physical Activity Behavior			
Days activity monitor worn	6.2 (0.7)	6.1 (0.5)	−0.10
Steps/day​​	6460 (2409)	6868 (2231)	0.18
Steps/day at cadence > 100 steps/min​​	1587 (991)	1714 (1054)	0.12
Stepping Time at > 100 steps/day (min) ​	14.3 (8.7)	15.6 (9.3)	0.15
Stepping Time (min/day)​​	84.6 (28.8)	89.5 (26.8)	0.18
Standing Time (min/day)	205.2 (56.6)	205.6 (59.2)	0.01
Sitting Time(min/day)	484.3 (72.7)	471.5 (94.1)	−0.15
Sitting Time > 60 min (min/day)​​	138.7 (77.1)	126.5 (75.3)	−0.16
Activity Score (MET.h)​​	32.9 (1.1)	33.1 (1.0)	0.18
Eating Behaviors			
Daily Kilocalories	2162 (668)	2038 (576)	−0.20
Total Vegetables (out of 5)	2.8 (1.7)	2.9 (1.4)	0.11
Greens and Beans (out of 5)	2.1 (2.3)	1.4 (1.8)	−0.36
Total Fruit (out of 5)	2.0 (2.1)	1.9 (2.2)	−0.02
Whole Fruit (out of 5)	2.2 (2.3)	2.1 (2.3)	−0.07
Whole Grains (out of 10)	3.1 (3.4)	2.6 (2.5)	−0.15
Total Dairy (out of 10)	5.8 (3.3)	6.4 (2.7)	0.19
Total Proteins (out of 5)	4.0 (1.2)	4.9 (0.6)	0.57
Seafood/Plant Protein (out of 5)	1.8 (2.1)	1.9 (2.1)	0.06
Fatty Acids (out of 10)	5.7 (2.9)	4.2 (3.1)	−0.53
Sodium (out of 10)	4.3 (3.3)	4.1 (2.5)	−0.06
Refined Grains (out of 10)	5.9 (3.4)	5.9 (2.9)	0.02
Saturated Fat (out of 10)	4.6 (3.0)	4.0 (3.1)	−0.17
Added Sugars (out of 10)	7.6 (2.4)	7.1 (2.8)	−0.21
Total HEI Score (out of 100)	52.1 (13.3)	49.1 (11.9)	−0.23
Sleep Behaviors			
Total Sleep Time (hours)	7.1 (0.7)	7.1 (0.6)	0.08
Time in Bed (hours)	7.9 (0.7)	7.9 (0.6)	0.03
Onset Latency (hours)	0.2 (0.1)	0.2 (0.0)	0.72
Sleep Efficiency (%)	86.5 (4.7)	86.9 (4.1)	0.09
WASO (hours)	0.7 (0.3)	0.7 (0.2)	0.13
Number of Awakenings	8.0 (3.3)	8.9 (3.4)	0.27

We observed large pre-post effects for cancer-related health outcomes in the expected direction including fatigue (d = −0.86), sleep disturbance (d = −0.95) and global health scores (d = 0.99) (see Table 5). Medium-size effects in the expected direction were observed for social roles (d = −0.68) and anxiety (d = −0.60). Small effects in the expected direction were observed for depression (d = −0.24), physical function (d = −0.31), and pain intensity (d = −0.35). A large effect in the unexpected direction was observed for pain interference (d = 1.01).

**Table 5:** Cancer-related health outcomes at baseline and post-intervention (*N* = 30)

PROMIS Variable	Baseline Mean (SD)​	Post-Intervention Mean (SD)	Cohen’s d
Physical Function (out of 20)​	5.7 (1.8)	5.2 (1.4)	−0.31
Anxiety (out of 20)​	7.9 (2.2)​	6.6 (2.1)	−0.60
Depression (out of 20)	6.0 (2.2)​	5.5 (2.0)	−0.24
Fatigue (out of 20)​	11.6 (2.9)	9.1 (2.9)	−0.86
Sleep Disturbance (out of 20)	11.2 (2.5)​	9.0 (2.1)	−0.95
Social Roles (out of 20)​	8.6 (2.7)	6.8 (2.6)	−0.68
Pain Interference (out of 20)​	7.5 (2.7)	9.9 (2.0)	1.01
Pain Intensity (out of 10)​	2.6 (1.8)	2.0 (1.6)	−0.35
Global Health (out of 10)​*	6.0 (1.1)	7.0 (0.9)	0.99

*Higher score indicates healthier status. For all other outcomes, lower scores indicate healthier status

## Discussion

This pilot study provides preliminary support for the feasibility and acceptability of a remotely delivered, theory-guided lifestyle intervention for young and middle-aged cancer survivors. The intervention achieved low attrition rate, high intervention adherence rates, and strong participant satisfaction. Preliminary pre-post analyses suggest (1) medium to large effects for several targeted psychosocial outcomes; (2) trivial to medium effects for targeted health behaviors; (3) small-large effects for several cancer-related health outcomes. Collectively, the findings suggest this intervention can be effectively delivered to survivors of cancer living across the country in both urban and rural areas.

### Feasibility 

Our enrollment rate (49%) fell just below the 50% benchmark and our retention rate (83%) exceeded expectations and compared favorably with similar trials (e.g., 74% in Price et al. [47] and 71% in Wood et al. [48]). The most effective recruitment method was through electronic health records, while mass emails and social media were less successful. These data support prioritizing clinical recruitment pathways in future studies and suggest the need to better align marketing materials with survivor motivations.

Our adherence rates also indicate strong participant engagement. We sought to maintain a 70% adherence rate for all three intervention features (health coaching, health education, wearable monitor wear). We exceeded our goals in each of these areas (≥ 84% across all components) which could be due to the personalized nature of health coaching and the flexible delivery format. The use of trained undergraduate student health coaches enrolled in a service-learning internship and the Healthie platform facilitated both fidelity and scalability. Comparing intervention adherence rates with other studies is difficult considering the wide variability in the dose of health coaching offered across interventions. Still, our findings are similar to those of Price et al. who found participants attended 95% of all available sessions in their 12-week tele-health intervention [47].

### Acceptability

 We sought for at least 75% of participants responding either “agree” or “strongly agree” on all four items of program satisfaction. We exceeded this goal for all four items which suggests the intervention was well received. Satisfaction with the Healthie mobile app and the health education videos was modest. Future versions should refine the app experience and consider enhancing or personalizing video content to maintain engagement.

### Psychosocial Mechanisms 

Medium-large improvements were observed in behavioral self-regulation skills, identity, and habits—key mechanisms aligned with the M-PAC theory and associated with initiation and maintenance of health behaviors. These findings underscore the value of using a theoretical model to target behavior change mechanisms and support the integration of reflection and identity-building activities in coaching sessions. Small effects observed for affective attitudes, perceived capability, and intentions suggest the intervention is less effective for improving these outcomes. This finding may be due to ceiling effects whereby participants entering the intervention had high baseline scores in these areas.

### Health Behaviors 

Few notable effects were observed for measured health behaviors including physical activity, diet, or sleep behaviors. This may reflect both the heterogeneity of baseline behavior levels and the person-centered nature of coaching, which encourages participants to identify and pursue their own behavioral goals. Specifically, while participants were encouraged to modify their PA, diet, and sleep, many chose to target only one behavior, perhaps diluting sample-level effects. Additionally, some participants entered the study already meeting behavioral guidelines for a particular behavior, thus limiting room for improvement (e.g., 7 participants had HEI scores > 60; several met physical activity and sleep targets). Future studies might classify outcomes as “meeting vs. not meeting recommendations” or stratify analysis by baseline status to account for ceiling effects and better capture the effects of the intervention.

### Cancer-Related Health Outcomes 

Medium-large changes in fatigue, sleep disturbance, and global health scores suggest a broader impact of the intervention beyond psychosocial and behavioral outcomes alone. These improvements may be interrelated (e.g., better sleep quality may contribute to reduced fatigue and improved participation in daily roles) [49]. It is also possible that changes in psychosocial outcomes could be driving changes in cancer-related outcomes (e.g., improvements in behavioral regulation skills drive improvements in perceived global health). Future studies that are sufficiently powered to explore these relations are encouraged to conduct mediation analyses to better understand such relations.

Interestingly, participants reported large pre-post increases in pain interference. It is unclear whether this change was caused by the intervention or external factors. This is difficult to decipher as previous studies have found that up to 36% of survivors of cancer experience chronic pain [50]. Additionally, all treatment modalities have potential to cause pain, and pain tends to fluctuate over time among survivors. Regardless, future trials may require more frequent pain assessments that inquire about the source of pain. Such assessments should focus on determining the effect of the behavioral intervention on pain presence and intensity.

### Limitations and Strengths 

The findings of this study should be interpreted with caution due to a few notable limitations. The modest sample size and single-arm design limited our ability to fully test the efficacy of the intervention. Our measure of diet (a single 24-hour recall at baseline and post-intervention) is likely not sufficient to estimate typical dietary behaviors and should be interpreted with caution. Future trials should consider multiple, non-consecutive recalls to more accurately capture typical dietary behaviors. Our participants were majority female, non-Hispanic White, and well-educated, which limits the overall generalizability of our findings. Strengths of the study include successfully delivering the intervention completely remotely using a combination of telehealth software and wearable monitors. We also delivered the intervention using a service-learning model that provided emerging health coaches with critical experiences while also providing a free health service to cancer survivors living across a wide region of the U.S. These features allowed us to deliver the intervention at scale and to expand the reach of the intervention. These are important considering lifestyle behavior change programs are limited in community settings, especially in rural areas which often suffer from healthcare shortages. The study design was informed by the ORBIT model [30] and the intervention design was informed by the M-PAC theory [31]. Finally, physical activity and sleep were both measured objectively using research grade objective monitors.

## Conclusion

This intervention demonstrated strong feasibility and acceptability among young and middle-aged cancer survivors. Observed changes in psychosocial mechanisms, health behaviors, and cancer-related health outcomes suggest the intervention may support meaningful improvements in survivors’ overall well-being. Based on the ORBIT model, a logical next step is to conduct a larger, randomized phase II trial with a control group and sufficient power to test specific outcomes. Given the individualized nature of health coaching, future studies should consider flexible outcome measures aligned with participant priorities—such as improvements in health-related quality of life.

## Data Availability

The datasets generated during and/or analyzed during the current study are available from the corresponding author on reasonable request.
